# Hybrid de novo transcriptome assembly of poinsettia (*Euphorbia pulcherrima* Willd. Ex Klotsch) bracts

**DOI:** 10.1186/s12864-019-6247-3

**Published:** 2019-11-27

**Authors:** Vinicius Vilperte, Calin Rares Lucaciu, Heidi Halbwirth, Robert Boehm, Thomas Rattei, Thomas Debener

**Affiliations:** 1 Institute of Plant Genetics, Leibniz Universität Hannover, 30419 Hannover, Germany; 2 Klemm + Sohn GmbH & Co., 70379 Stuttgart, KG Germany; 3 Department of Microbiology and Ecosystem Science, University of Vienna, 1090 Vienna, Austria; 4 Institute of Chemical, Environmental and Bioscience Engineering, Technische Universität Wien, 1060 Vienna, Austria

**Keywords:** Poinsettia (*Euphorbia pulcherrima*), RNA-Seq, Anthocyanin, Hybrid de novo transcriptome, Bract coloration

## Abstract

**Background:**

Poinsettia is a popular and important ornamental crop, mostly during the Christmas season. Its bract coloration ranges from pink/red to creamy/white shades. Despite its ornamental value, there is a lack of knowledge about the genetics and molecular biology of poinsettia, especially on the mechanisms of color formation. We performed an RNA-Seq analysis in order to shed light on the transcriptome of poinsettia bracts. Moreover, we analyzed the transcriptome differences of red- and white-bracted poinsettia varieties during bract development and coloration. For the assembly of a bract transcriptome, two paired-end cDNA libraries from a red and white poinsettia pair were sequenced with the Illumina technology, and one library from a red-bracted variety was used for PacBio sequencing. Both short and long reads were assembled using a hybrid de novo strategy. Samples of red- and white-bracted poinsettias were sequenced and comparatively analyzed in three color developmental stages in order to understand the mechanisms of color formation and accumulation in the species.

**Results:**

The final transcriptome contains 288,524 contigs, with 33% showing confident protein annotation against the TAIR10 database. The BUSCO pipeline, which is based on near-universal orthologous gene groups, was applied to assess the transcriptome completeness. From a total of 1440 BUSCO groups searched, 77% were categorized as complete (41% as single-copy and 36% as duplicated), 10% as fragmented and 13% as missing BUSCOs. The gene expression comparison between red and white varieties of poinsettia showed a differential regulation of the flavonoid biosynthesis pathway only at particular stages of bract development. An initial impairment of the flavonoid pathway early in the color accumulation process for the white poinsettia variety was observed, but these differences were no longer present in the subsequent stages of bract development. Nonetheless, *GSTF11* and *UGT79B10* showed a lower expression in the last stage of bract development for the white variety and, therefore, are potential candidates for further studies on poinsettia coloration.

**Conclusions:**

In summary, this transcriptome analysis provides a valuable foundation for further studies on poinsettia, such as plant breeding and genetics, and highlights crucial information on the molecular mechanism of color formation.

## Background

The poinsettia, *Euphorbia pulcherrima* Willd. ex Klotsch, also known as Nochebuena or Christmas Star, is one of the most important ornamental potted plants around the globe. The species is native to Mexico [76] and belongs to the family Euphorbiaceae and genus *Euphorbia*, with the latest estimate containing around 2000 species and representing one of the largest genera within angiosperms [31]. The species is known by its red bract coloration, which is due to the accumulation of anthocyanin pigments. Anthocyanins are a class of flavonoid secondary metabolite compounds [48] which provide orange to blue colors to flowers, seeds, fruits and other vegetative tissues in plants [72]. Moreover, they have multiple functional roles in plant-environment interactions, such as light protection and antioxidants, chelating agents for metals [43], as well as protection against biotic and abiotic stresses [2, 19]. The molecular mechanism involved in anthocyanin biosynthesis has been extensively described for several species [59], but only scarce information is currently available for poinsettia [30, 57].

In ornamental poinsettia, there is a coexistence of green, reddish, and red leaves/bracts [54] in the same plant, which implies a constant regulation of the anthocyanin and adjacent pathways throughout the bract development process. A bract is a modified or specialized leaf, often associated with a reproductive structure such as a flower or inflorescence. In poinsettia, bract axillary buds differentiate into flowers [36] under short day conditions, which is accompanied by the development and coloration of bracts, thus indicating that the anthocyanin metabolism is regulated by photoperiodism [34]. The color range in poinsettia varieties is obtained either through classical breeding (crossing) or mutagenic breeding (radiation), thus generating a spectrum of bract colors, such as pink, marble (pink center surrounded by white margins) and white/creamy. The pink coloration in pink and marble bracts are due to periclinal chimeric structures [55], while the reason for white/creamy coloration remains uncertain. Since the expression of all structural genes and the related enzyme activities involved in the formation of red anthocyanin pigments can be determined, the appearance of acyanic (uncolored) varieties is here referred to as the ‘*white paradox*’. The elucidation of such mechanisms is extremely valuable for this crop since the production of plants with bright and/or different colors is a key aspect for breeding and consumer acceptance [30]. Despite the popularity of poinsettia, information about its genome and transcriptome have not been generated yet. Transcriptome assemblies are very useful in elucidating the major transcripts and isoforms involved in pigmentation pathways, as well as their expression profiles under specific conditions [3, 24, 47, 96].

De novo transcriptome assemblies still represent a challenge for non-model plant species, where the general approach relies on the use of short cDNA sequences (such as Illumina technology). Some of the issues faced are related to the sensitivity of alignment errors due to paralogs and multigene families, production of artefactual chimeras and fragmented genes, and potentially misestimated allelic diversity [17]. The recent use of PacBio technology has generated an improvement in various plant transcriptomes [5, 80, 87] since it is able to generate full-length transcripts without the need of assembly algorithms. Nevertheless, long reads generated by the PacBio technology show an error rate of 13–15% [6] and, therefore, deep sequencing is required to correct the errors based on base coverage. As an alternative, a hybrid assembly approach (combining short and long reads) could be implemented to achieve similar results. Although still scarce, some methods have shown the applicability and usefulness of this approach to improve transcriptome annotations [25, 56, 84].

With the aim of generating valuable information on molecular aspects of poinsettia, we have assembled and functionally annotated a de novo bract transcriptome for the species. In addition, we also underlined and characterized the regulation of the main pathways involved in the transition of green leaves to colored bracts. Lastly, we characterized the main differences between red- and white-bracted poinsettia varieties, focusing on the flavonoid and adjacent pathways that are involved in pigment accumulation in plant tissues. Due to tissue-specific expression and the difficulty of recovering low expressed transcripts, the de novo assembled transcriptome is not expected to represent the entire range of transcripts of the species; nevertheless, the successful assembly of different isoforms and the differential expression analysis enabled a first insight into the *white paradox*.

## Results

### De novo assembly and functional annotation of the poinsettia bract transcriptome

In order to create a representative transcriptome for poinsettia bracts, cDNA libraries of the variety pair Christmas Feelings (red) and Christmas Feelings Pearl (white) were sequenced using the Illumina NextSeq500 system. In addition, a full-length cDNA library, from the Vintage variety (red), was sequenced using the PacBio Sequel System. After quality control and data cleaning, 36,989,889 and 35,404,728 Illumina reads were generated for the red and white varieties, respectively, with an average proportion of 77.4% clean reads for the libraries. The Iso-Seq pipeline v3.0 was applied to the PacBio dataset and, after sequence classification, clustering, and quality control, a total of 30,768 high-quality full-length transcripts were generated (Table 1).

**Table 1:** Summary of Illumina and PacBio sequencing

Illumina sequencing			
Variety	Total number of reads	Remained reads after rRNA removal	Remained reads after quality trimming (QV ≥ 20)
Christmas Feelings	46,734,786	43,267,294	36,989,889
Christmas Feelings Pearl	46,772,696	42,704,780	35,404,728
PacBio sequencing			
Variety	Total number of CCS	Number of FLNC reads	Number of polished transcripts
Vintage	72,202	52,077	30,768

We mapped the Illumina post-processed reads to the PacBio transcripts to assess their completeness and to verify if they represent a significant portion of the transcriptome. The distribution of average coverage over the full-length transcripts is shown in Additional file 1. The majority of the full-length transcripts were covered by both Illumina datasets. Out of 30,768 full-length transcripts, 1987 were not covered by the Illumina reads from the red variety, while 1808 were not covered by the reads from the white variety. Moreover, the overall mapping rate was 60 and 58% of read pairs for the red and white varieties, respectively. These results imply that the PacBio transcripts did not seem to capture the majority of the bract transcriptome of poinsettia, thus not suitable to be used as the only dataset for our transcriptome. To overcome that, a hybrid de novo assembly strategy was applied.

The Trinity tool was used to perform the de novo assembly with both Illumina and PacBio post-processed reads. The final assembly contains 288,524 contigs belonging to 138,702 genes, with a total of 257,619,354 assembled bases, GC content of 38.23% and an N50 of 1488. To evaluate the quality and coverage of the assembled transcripts, the Illumina reads were re-mapped to the final transcriptome using bowtie2. The re-mapping ratio was 83 and 81% for Christmas Feelings and Christmas Feelings Pearl, respectively. Next, the assembled transcripts were annotated against TAIR10 and SwissProt databases. From 288,524 total contigs assembled, 78,350 (27.1%) showed annotation against the SwissProt database, while 95,900 (33.2%) of them showed homology to *A. thaliana* transcripts (TAIR10), both using an *E-value* < 1E-20. Due to the higher number of retrieved annotations, we used the data from TAIR10 for further analyses. A total of 14,623 *A. thaliana* homologous transcripts were identified in our transcriptome (Additional file 2), with 6105 showing a length coverage between 90 and 100% (Additional file 3). Functional annotation and Gene Ontology (GO) terms were retrieved using the online tool agriGO. Out of the 14,623 different *A. thaliana* homologous transcripts, 13,809 (94.4%) were assigned to one or more GO terms. On the other hand, 814 homologous transcripts (representing 6261 transcripts in our transcriptome) could not be assigned to GO terms.

**Fig. 1:**
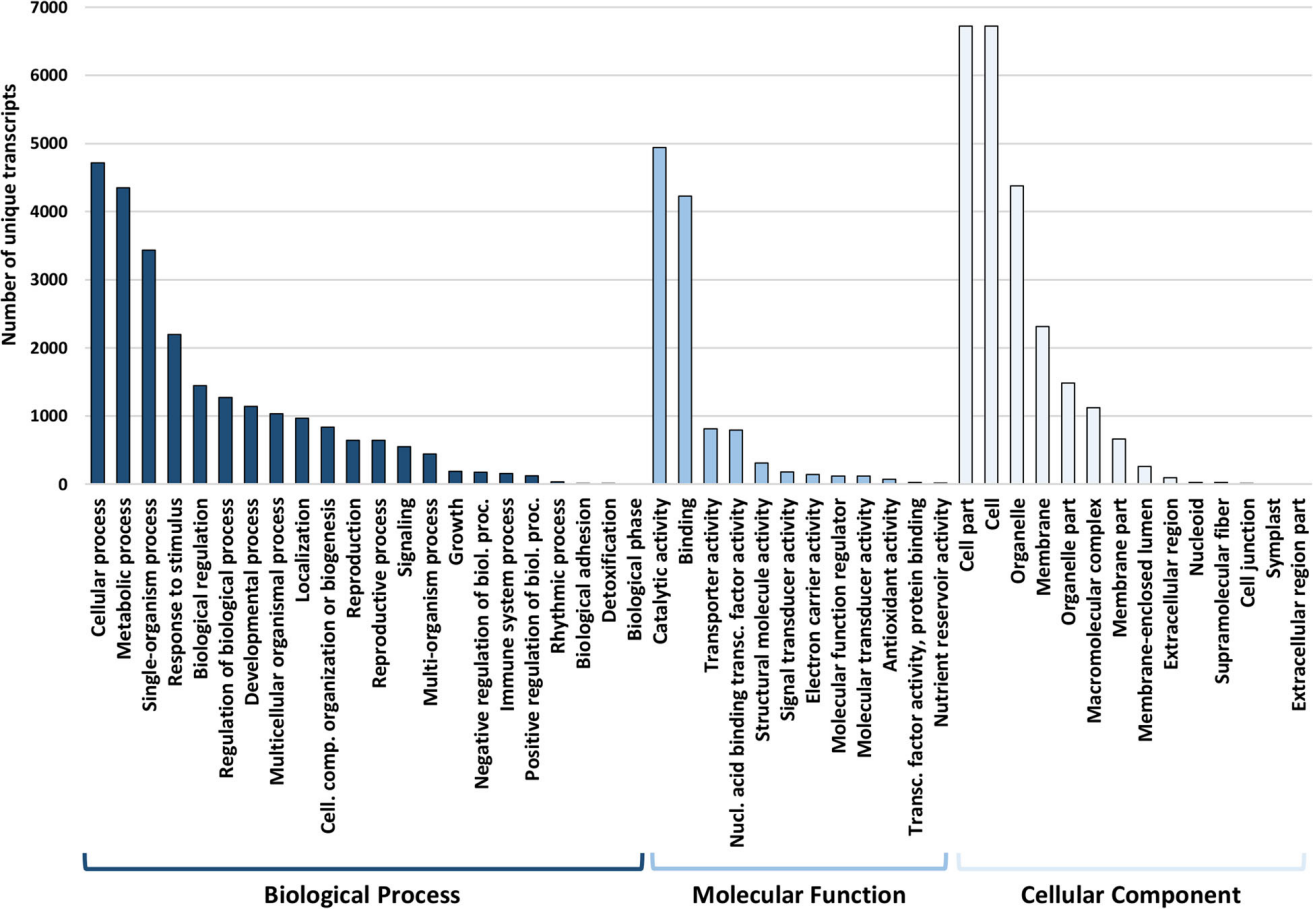
Functional annotation of the assembled transcripts from poinsettia bracts. Annotated transcripts were assigned to gene ontology terms and classified as biological process, molecular function, and cellular component

In total, 13,809 unique transcripts were functionally characterized in 48 subcategories and grouped in three main groups: biological process (22 subcategories), molecular function (12) and cellular component (14), with several transcripts annotated with multiple GO terms (Fig. 1). Within the biological process category, cellular process (4716) and metabolic process (4348) were prominent, indicating a higher number of genes involved in important metabolic activities. In the molecular function category, the majority of the GO terms were grouped into catalytic activity (4941) and binding (4225), followed by transporter (811) and nucleic acid binding (791) activities. For the cellular component category, 6721 GO terms were assigned to both cell and cell part, and, together with organelle (4376) and membrane (2314), represent the dominant transcripts in this category.

Several genes related to the flavonoid biosynthetic pathway were identified in our bract transcriptome. The annotation against the TAIR10 database revealed 127 transcripts belonging to 23 known flavonoid-related structural genes and 24 transcripts belonging to six flavonoid-related transcription factors (Table 2). The genes with the highest number of identified transcripts were *Flavone 3′-O-methyltransferase 1* (15), *Hydroxycinnamoyl-CoA shikimate transferase* (12) and *Dihydroflavonol 4-reductase* (11). On the other hand, *Phenylalanine ammonia-lyase 4*, *Flavanone 3-hydroxylase* and *TTG1 Transducin/WD40 repeat-like* were the only genes that contained a single transcript. Similar genes were identified in another poinsettia transcriptome, also with a high number of transcripts assigned to different genes [30]. Moreover, it is important to note that, due to the lack of an available genome, poinsettia specific transcripts might not have been identified and, therefore, a higher number of transcripts might be involved in the flavonoid pathway. The expression of several flavonoid-related genes found in our transcriptome, as well as previous metabolite profiling studies [30, 68], implies that poinsettia bract pigmentation is achieved through the regulation of those genes and further accumulation of flavonoid compounds.

**Table 2:** List of flavonoid biosynthesis related genes identified in the poinsettia bract transcriptome

	*A. thaliana* orthologous	Gene	Enzyme name	# of transcripts identified
Structural genes	AT2G37040.1	*PAL1*	Phenylalanine ammonia-lyase 1	4
	AT3G53260.1	*PAL2*	Phenylalanine ammonia-lyase 2	5
	AT3G10340.1	*PAL4*	Phenylalanine ammonia-lyase 4	1
	AT2G30490.1	*C4H*	Trans-cinnamate-4-hydroxylase	8
	AT1G51680.1	*4CL1*	4-coumarate: CoA ligase 1	3
	AT3G21240.1	*4CL2*	4-coumarate: CoA ligase 2	5
	AT1G65060.1	*4CL3*	4-coumarate: CoA ligase 3	4
	AT3G21230.1	*4CL5*	4-coumarate: CoA ligase 5	2
	AT5G13930.1	*CHS*	Chalcone synthase	4
	AT5G48930.1	*HCT*	Hydroxycinnamoyl-CoA shikimate transferase	12
	AT5G05270.1	*CHI3*	Chalcone-flavonone isomerase 3	2
	AT3G55120.1	*CHI1*	Chalcone-flavonone isomerase 1	4
	AT3G51240.1	*F3H, FHT*	Flavanone 3-hydroxylase	1
	AT5G07990.1	*F3’H*	Flavonoid 3′-hydroxylase	10
	AT5G08640.1	*FLS1*	Flavonol synthase 1	5
	AT5G42800.1	*DFR*	Dihydroflavonol 4-reductase	11
	AT4G22880.1	*LDOX, ANS*	Leucoanthocyanidin dioxygenase, Anthocyanin synthase	3
	AT5G17050.1	*UGT78D2*	UDP-glucosyl transferase 78D2	10
	AT3G29590.1	*5MAT*	Anthocyanidin 5-*O*-glucoside-6″-*O*-malonyltransferase	2
	AT1G61720.1	*ANR*	Anthocyanidin reductase	10
	AT5G54160.1	*OMT1*	Flavone 3′-*O*-methyltransferase 1	15
	AT3G59030.1	*TT12*	TRANSPARENT TESTA 12	5
	AT3G03190.1	*GSTF11*	Glutathione S-transferase	4
Transcription factors	AT1G63650.1	*EGL1*	Transcription factor EGL1	7
	AT1G66370.1	*MYB113*	MYB domain protein 113	2
	AT2G47460.1	*MYB12*	MYB domain protein 12	4
	AT5G35550.1	*TT2*	Transcription factor TT2 (MYB123)	6
	AT4G09820.1	*TT8*	Transcription factor TT8 (bHLH42)	4
	AT5G24520.1	*TTG1*	TRANSPARENT TESTA GLABRA 1	1

### Transcriptome completeness and comparison to related species

A transcriptome represents the complete set and quantity of transcripts from a specific stage of development or physiological condition [78]. By relying on bract material to assemble the transcriptome of poinsettia, transcripts specific to other plant tissues, e.g. root and stem, could be missing in bracts. For a better overview of the completeness of the poinsettia bract transcriptome generated in the present study, publicly available sequences from root, stem and leaf tissues of *Euphorbia pekinensis* were retrieved and individual transcriptomes for each tissue were assembled and annotated. Based on the annotation against the TAIR10 database, tissue-specific transcripts were observed for each of the *E. pekinensis* transcriptomes. A total of 2149 Arabidopsis homologous proteins from all three *E. pekinensis* transcriptomes were not present in our poinsettia bract transcriptome. From these proteins, 317 were uniquely present in the leaf transcriptome, while 346 and 235 homologous proteins were uniquely detected in root and stem transcriptomes, respectively. On the other hand, 1262 Arabidopsis homologous proteins present on the bract transcriptome were not detected in any of the *E. pekinensis* transcriptomes.

The BUSCO pipeline, which is based on near-universal orthologous gene groups, was applied to assess the completeness of the newly assembled poinsettia bract transcriptome, as well as the *E. pekinensis* transcriptomes. This pipeline permits to assess the completeness of transcriptomes based on evolutionarily informed expectations of gene content. Therefore, it enables like-for-like quality comparisons of different data sets (e.g. transcriptomes) [83]. From a total of 1440 BUSCO (embryophyta_odb9 database) groups searched, the poinsettia bract transcriptome showed 1115 (77%) categorized as complete (595 (41%) as single-copy and 520 (36%) as duplicated), 139 (10%) as fragmented and 186 (13%) as missing BUSCOs (Table 3). The BUSCO results for the *E. pekinensis* transcriptomes are also shown in Table 3.

**Table 3:** Completeness assessment of *E. pulcherrima* and *E. pekinensis* transcriptomes by the BUSCO pipeline

Species - Tissue	Complete BUSCOs		Fragmented BUSCOs	Missing BUSCOs
	Single-copy	Duplicated		
*E. pulcherrima* - Bract	41.3%	36.1%	9.7%	12.9%
*E. pekinensis* - Leaf	31.3%	50.1%	8.9%	9.7%
*E. pekinensis* - Root	32.0%	46.5%	10.2%	11.3%
*E. pekinensis* - Stem	36.3%	41.0%	9.1%	13.6%

When comparing the completeness of the poinsettia bract with the tissue-specific transcriptomes from *E. pekinensis*, we noticed that the number of complete BUSCOs is comparable in all transcriptomes, but with poinsettia showing a lower percentage of duplicated ones. Additionally, the number of fragmented and missing BUSCOs also showed similar percentages. Out of 186 missing BUSCOs in the bract transcriptome (12.9%), 136 of them were identified in at least one of the *E. pekinensis* transcriptomes, with 16 exclusively present in the leaf transcriptome and another 16 exclusively present in the root transcriptome. The most abundant orthologs among those groups belonged to the Pentatricopeptide repeat (PPR) superfamily protein. In addition, 50 ortholog groups are equally missing in all four transcriptomes, with the majority of them also belonging to PPR superfamily protein groups. On the other hand, 171 ortholog groups present in the bract transcriptome were completely absent from all three *E. pekinensis* transcriptome. The list of missing BUSCO orthologs for one or more of the transcriptomes is available in Additional file 4. All in all, the BUSCO analysis shows that tissue-specific orthologs might be absent in our poinsettia bract transcriptome. Nevertheless, a high level of transcriptome completeness was observed and thus enables us to reliably use the data for further analyses.

### Differential expression analysis of poinsettia bracts

To understand the dynamics of gene expression in different stages of bract and color development of poinsettia, RNA-Seq libraries from three independent biological replicates of the Christmas Feelings and Christmas Feelings Pearl varieties, sampled at three developmental stages (Stage 1 - S1, Stage 2 - S2 and Stage 3 - S3), were sequenced for transcriptome analysis. In total, 927,560,033 million raw reads with a length of 75 bp were obtained and, after quality trimming and rRNA removal, an average of 91.6% reads remained available. The overall mapping of the datasets against the poinsettia bract transcriptome was 92.9% (Additional file 5). In addition, a high correlation between biological replicates (Pearson correlation) was observed, thus showing the reliability of the datasets (Additional file 6).

The RNA-Seq data from the three bract developmental stages were compared using two different approaches. First, we aimed to characterize the variation in gene expression between the different stages of bract development, regardless of the bract color. Hereof, we compared the six samples from S1 (three Christmas Feelings and three Christmas Feelings Pearl as independent biological replicates) against the six samples from S2, as well as S2 against S3. Secondly, we were interested in analyzing the differences between red and white bracts for each of the time points, especially those related to biosynthesis and accumulation of pigments. To this end, we compared the Christmas Feelings and Christmas Feelings Pearl varieties of each stage against each other.

### Characterization of the expression profiles of poinsettia bracts during three developmental stages

To characterize the gene regulation dynamics in the transition of green leaves to fully developed bracts, six independent biological replicates (three replicates from Christmas Feelings and three replicates from Christmas Feelings Pearl) for three bract developmental stages were analyzed. The pairwise comparison for the first transition point, between S1 and S2, showed significantly lower expression rates for 3743 transcripts in S2. A pathway enrichment analysis of the DEGs was performed and 39 GO terms were differentially enriched (False Discovery Rate (FDR) ≤ 0.05). The enriched pathways linked to major biological processes included: i) response to temperature stimulus (GO:0009266); ii) enzyme-linked receptor protein signaling pathway (GO:0007167); and iii) response to heat (GO:0009408). On the other hand, 2675 transcripts were higher expressed in the S2 samples. Pathway enrichment analysis showed that 22 GO terms were differentially enriched, with the major molecular functions enriched pathways being related to: i) catalytic activity (GO:0003824); ii) oxidoreductase activity (GO:0016491); and iii) peptidase activity (GO:0008233).

For the second transition point, S2 to S3, 4479 transcripts had significantly lower expression in S3. A total of 104 GO terms were differentially enriched, with the major biological processes being related to response to temperature stimulus (GO:0009266) and photosynthesis (GO:0015979). Additionally, 5253 transcripts showed higher expression in S3. Pathway analysis showed 71 GO terms differentially enriched, with transmembrane receptor signaling pathway (GO:0007169) and phenylpropanoid metabolic/biosynthetic processes (GO:0009698/GO:0009699) being the major biological processes differentially regulated. The lists of differentially expressed transcripts, as well as the enriched GO terms for all comparisons are available in Additional files 7 and 8, respectively.

Many genes involved in photosynthesis and phenylpropanoid related pathways were found to be differentially expressed between stages 2 and 3, and they were involved in distinct biological processes (Table 4). The list of individual genes involved in each biological process is available in Additional file 9. It has been shown that, during bract development in poinsettia, photosynthetic pigments are synthesized early and then replaced by different phenolic compounds [27, 36]. Thus, a significantly lower expression of genes related to photosynthesis, accompanied by a higher expression of flavonoid biosynthesis genes (phenylpropanoid pathway), was expected along with this transition.

**Table 4:** Differentially enriched photosynthesis- and phenylpropanoid-related pathways between stages 2 and 3 of poinsettia bract development

Down-regulated in stage 3			
GO term	Term description	Genes identified	FDR
GO:0015979	Photosynthesis	51	1.5E-10
GO:0006091	Generation of precursor metabolites and energy	42	0.0033
GO:0009657	Plastid organization	39	1.7E-07
GO:0019684	Photosynthesis, light reaction	34	3.5E-09
GO:0009658	Chloroplast organization	22	0.0087
GO:0009767	Photosynthetic electron transport chain	13	0.0054
GO:0045036	Protein targeting to chloroplast	10	0.0087
GO:0010027	Thylakoid membrane organization	10	0.0087
GO:0072598	Protein localization to chloroplast	10	0.0087
GO:0072596	Establishment of protein localization to chloroplast	10	0.0087
GO:0009668	Plastid membrane organization	10	0.0095
GO:0009773	Photosynthetic electron transport in photosystem I	8	0.0029
GO:0010207	Photosystem II assembly	7	0.0190
GO:0045038	Protein import into chloroplast thylakoid membrane	5	0.0100
Up-regulated in stage 3			
GO:0009698	Phenylpropanoid metabolic process	27	0.0490
GO:0009699	Phenylpropanoid biosynthetic process	25	0.0059

### Characterization of expression differences between red and white poinsettia varieties

**Fig. 2:**
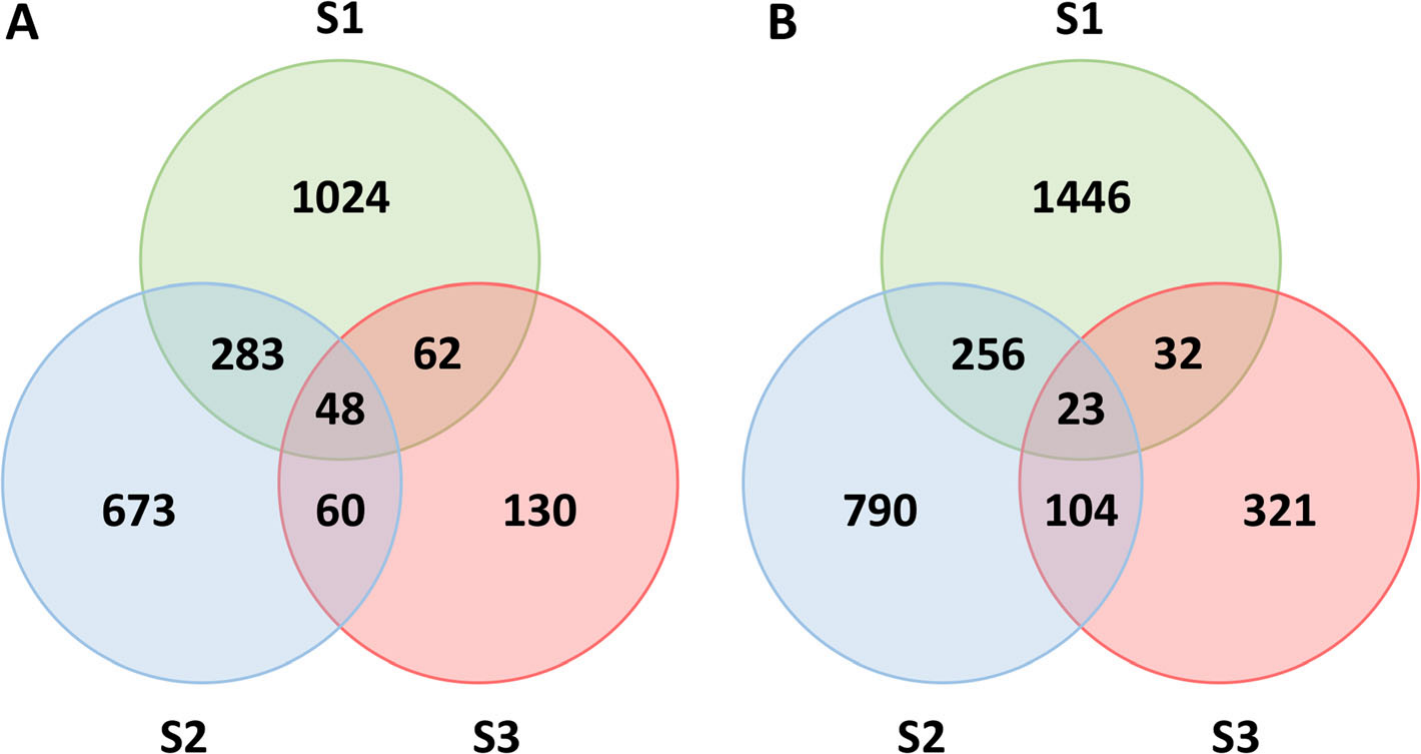
Venn diagram of the differentially regulated transcripts for the different bract developmental stages of poinsettia. **a** Transcripts with a lower expression in white bracts; **b** Transcripts with a higher expression in white bracts. S1, S2 and S3 = Stages 1, 2 and 3, respectively

For the characterization of the differences between Christmas Feelings and Christmas Feelings Pearl, three independent biological replicates were used for each of the varieties, and the comparison was performed for the three bract development stages. The pairwise comparison revealed 1204 transcripts with a lower expression in white bracts on the first stage, while only 130 were lower expressed on stage two and 673 on stage three (FDR ≤ 0.05). However, only 48 transcripts were equally lower expressed in white bracts for all stages (Fig. 2a). On the other hand, 1446 transcripts were higher expressed in white bracts on the first stage, whilst a lower number of higher expressed transcripts were detected on stages two and three (321 and 790, respectively). Nonetheless, 23 were commonly high expressed in white bracts in all stages (Fig. 2b).

Pathway enrichment analysis was performed for the low- and high-expressed transcripts in white bracts for each of the developmental stages. Low expressed transcripts in the white bracts were associated with numerous biological processes. For stage one, 21 GO terms were differentially enriched, with major biological processes, such as response to temperature stimulus/heat (GO:0009266/GO:0009408) and flavonoid biosynthetic/metabolic process (GO:0009813/GO:0009812), among those. On the second stage, 11 GO terms were differentially enriched, with phosphorylation (GO:0016310) and protein phosphorylation (GO:0006468) among the major enriched biological processes pathways. As for the last stage, 10 GO terms were differentially enriched, with multidimensional cell growth (GO:0009825) and plant-type cell wall modification (GO:0009827) among the enriched biological processes.

In the same way, various biological processes were linked with the higher expressed transcripts in the white bracts. For the first stage, a total of 99 GO terms were found to be differentially enriched, with photosynthesis (GO:0015979 - photosynthesis / GO:0019684 – photosynthesis, light reaction / GO:0009767 - photosynthetic electron transport chain) and abiotic stimulus (GO:0009416 - response to light stimulus / GO:0009314 - response to radiation / GO:0009409 – response to cold) among those enriched pathways. As for the second stage, high expressed transcripts were involved in 62 differentially enriched GO terms. The main biological processes with a differential regulation were response to stimulus (GO:0050896), response to stress (GO:0006950), as well as phenylpropanoid biosynthetic/metabolic processes (GO:0009699/ GO:0009698). Lastly, 31 enriched GO terms were associated with the higher expressed transcripts in stage three. The main enriched biological processes were response to wounding (GO:0009611) and jasmonic acid biosynthetic/metabolic processes (GO:0009695/GO:0009694). Moreover, several molecular functions related to transferase and glucosyltransferase/glycosyltransferase activities (GO:0016757/GO:0008194/GO:0046527) were also enriched. The lists of differentially expressed transcripts, as well as the enriched GO terms for all comparisons are available in Additional files 10 and 11, respectively.

**Fig. 3:**
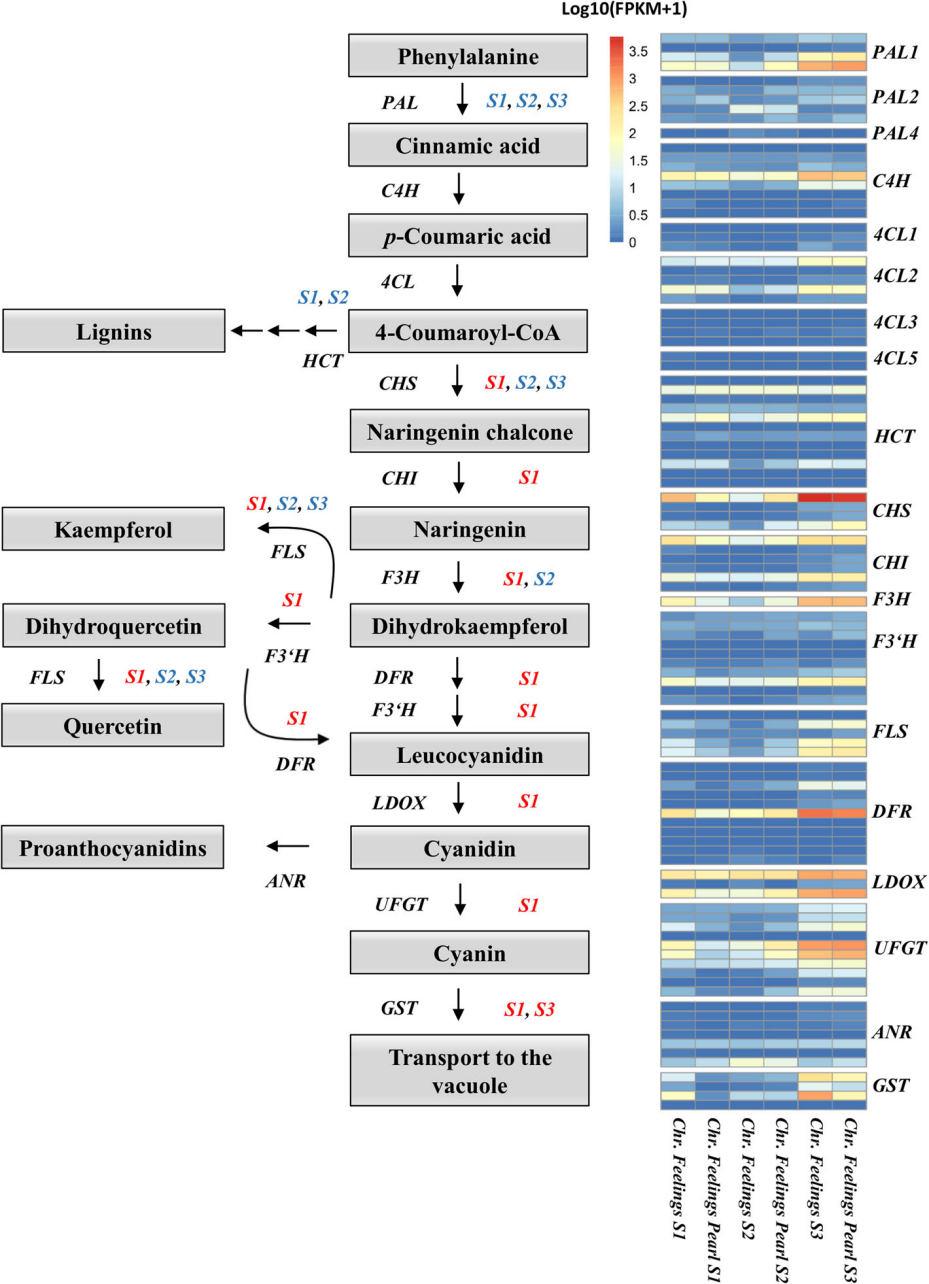
Anthocyanin biosynthetic pathway and expression of related genes during bract development in poinsettia varieties. (left) Differentially expressed genes (FDR ≤ 0.05) in the three stages of bract development are depicted by S1, S2 and S3 (Stages 1, 2 and 3, respectively) symbols next to the genes. Stages colored in red indicate a higher expression of the respective gene in the red poinsettia variety. Stages colored in blue indicate a higher expression of the respective gene in the white poinsettia variety. (right) Heatmap of the genes involved in each process of the pathway. Gene expression is represented by Log10(FPKM+ 1). FPKM = Fragments per kilobase per million*.* For gene abbreviations refer to Table 2

To further investigate possible differences in flavonoid biosynthesis genes, we analyzed the differentially expressed genes belonging to flavonoid metabolic process (GO:0009812) for each of the bract developmental stages between red and white poinsettia varieties. The main genes involved in the flavonoid biosynthesis and their difference in expression for each of the bract developmental stages are shown in Fig. 3. For the first stage of bract development, a total of 13 flavonoid-related genes showed differences in expression rates between red and white varieties, with 11 of them being lower expressed in the white variety (*CHS*, *CHI*, *F3H* (synonym: *FHT*), *F3’H*, *FLS1*, *DFR*, *LDOX*, *UFGT, MYB12, MYB113,* and *GSTF11*), while two of them showed a higher expression (*HCT* and *PAL2*). On the second stage, *PAL1*, *PAL2*, *HCT, CHS,* and *F3H* showed a higher expression in the white variety. For the last stage of bract development, five genes displayed differential expression between red and white varieties, with *GSTF11* being low expressed in the white variety, while *CHS*, *FLS*, *PAL2,* and *BEN* showed higher expression.

Two genes related to flavonoid biosynthesis showed antagonistic expression patterns along the bract development stages. *CHS* was lower expressed in white samples at the first stage, whereas in the second and third stages its expression was higher in white samples. As previously shown (Table 2), four transcripts were annotated as *CHS* in our bract transcriptome (here named *CHS1* to *CHS4*). *CHS1* was low expressed in the white variety in the first stage, but higher expressed in the second stage. In addition, *CHS2* was higher expressed in the white variety in the second and third stages. Similar results were identified for *FLS*, where five different transcripts were annotated as this gene in our transcriptome (here named *FLS1* to *FLS5*). *FLS1* and *FLS2* were lower expressed in white varieties on the first stage, while *FLS4* showed a higher expression in the last stage. Thus, the expression of some enzymes related to flavonoid biosynthesis might be driven by the complementary expression of multiple isoforms.

### Validation of gene expression patterns by RT-qPCR validation

**Fig. 4:**
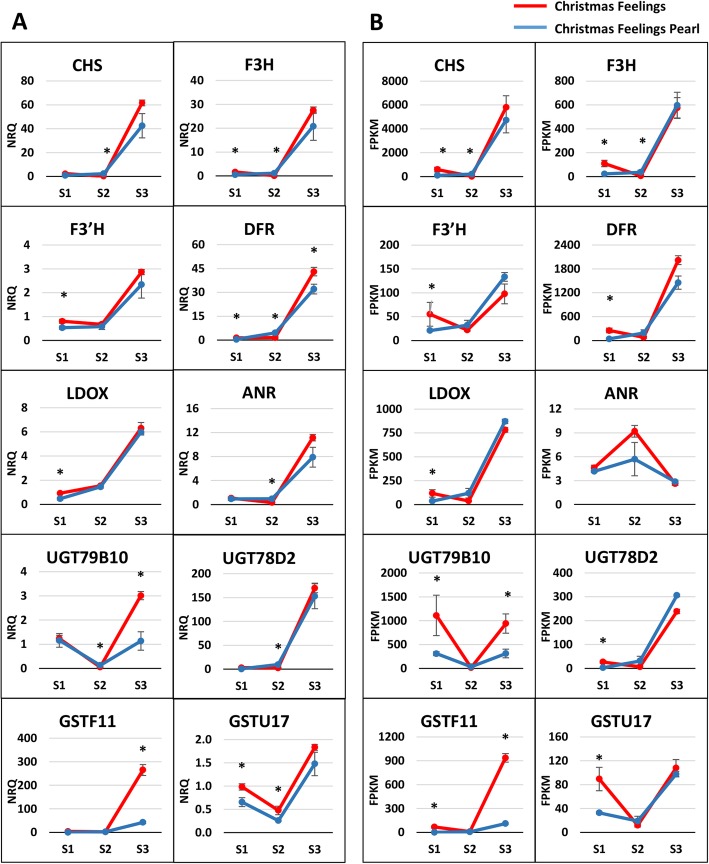
Expression profiles of anthocyanin-related genes for three developmental stages of poinsettia bracts. **a** RT-qPCR expression profiles of 10 anthocyanin related genes for the varieties Christmas Feelings and Christmas Feelings Pearl in three stages of bract development**. b** RNA-Seq expression profiles of 10 anthocyanin related genes for the varieties Christmas Feelings and Christmas Feelings Pearl in three stages of bract development. S1, S2, S3 = Stages 1, 2 and 3, respectively. Vertical bars indicate standard errors. ‘*’ symbol indicates significant differences for that specific stage for *p* ≤ 0.05. FPKM = Fragments per kilobase per million. NRQ = Normalized relative quantity. For gene abbreviations refer to Table 2

To further verify the expression profiles in the Illumina sequencing analyses, 10 transcripts were selected for RT-qPCR using the Christmas Feelings and Christmas Feelings Pearl varieties for each of the developmental stages used for RNA-Seq. The same biological triplicates used for RNA-Seq plus two extra independent biological samples were used for the RT-qPCR reactions. The selected genes are known to be part of the flavonoid and anthocyanin pathways in plants: *CHS*, *F3H*, *F3’H*, *DFR*, *ANR*, *LDOX*, *UGT79B10*, *UGT78D2*, *GSTF11,* and *GSTU17*. The normalized relative quantity (NRQ) obtained by RT-qPCR for each of the genes in the different time points and color bracts is shown in Fig. 4a. NRQ values were calculated relative to one of the biological replicates of the Christmas Feelings variety in stage 1 of bract development according to the Pffafl method and equations [60]. In addition, the RNA-Seq expression for each of the genes is shown in Fig. 4b.

Most of the genes analyzed by RT-qPCR showed a similar expression trend to the RNA-Seq data. *ANR* was the only analyzed gene that showed a completely different pattern of expression. The RT-qPCR primers were designed based on one of the transcripts annotated as an *A. thaliana ANR* homolog. However, several other transcripts have also been annotated as such (Table 2), with some of them showing distinct expression values among samples (data not shown), but none of them showing a differential expression on the RNA-Seq datasets. Moreover, other non-annotated transcripts might also have similarities to the designed primers and, therefore, might have been amplified in the RT-qPCR reaction. Nevertheless, these results indicate that the sequencing data produced in this study were accurate and reliable.

## Discussion

### Transcriptome assembly and annotation

Poinsettia is a widely popular ornamental plant, especially during the Christmas period, due to its red bract coloration. For the past years, a range of cultivars has been available, which exhibit differences mainly in height, growth habit, leaf size, and bract coloration. An understanding of the molecular mechanisms underlying bract development, particularly in color development and accumulation, will assist in the poinsettia breeding process to improve its ornamental value. However, scarce genetic information is available for the species. Complete genomes are only available for species from the same family, such as *Ricinus communis* [20], *Jatropha curcas* [66], *Manihot esculenta* [61] and *Hevea brasiliensis* [64], as well as some transcriptomes of *Euphorbia* species [9, 18, 32, 37, 62]. A recent transcriptome study has reported the assembly of 232,663 contigs arising from green leaf and red-turning bract of poinsettia [30], which is very similar to our transcriptome assembly (288,524 contigs). However, no functional annotation of the aforementioned transcriptome is available for comparison.

By applying the BUSCO pipeline, we confirmed that our transcriptome contains around 77% of the available ortholog groups at OrthoDB v9.1 [93]. Transcriptome studies with other plant species have shown a higher level of completeness (e.g. *Cinnamomum longepaniculatum* - 91% and *Noccaea caerulescens* - 90% [13, 90]), while others are similar to the ones in our transcriptome (e.g. *Camellia nitidissima* - 76% [101]). Moreover, different levels of BUSCO completeness were observed when comparing different tissues of the same species [8], thus indicating that tissue-specific transcripts may account for different coverages compared to what is expected for the complete gene space. Nonetheless, when comparing our results to the leaf, stem and root transcriptomes of *E. pekinensis* assembled in this study, comparable levels of BUSCO completeness were observed, as well as the presence of tissue-specific ortholog groups.

In this study, we used a hybrid de novo assembly strategy (Illumina and PacBio platforms) to generate a transcriptome for poinsettia bracts, where 95,900 out of 288,524 contigs were confidently annotated against *A. thaliana* transcripts (TAIR10). These represent a set of 14,623 distinct *A. thaliana* homologous transcripts. The 192,624 contigs without annotation might represent family- or species-specific transcripts, but also short and incomplete transcripts; nonetheless, they need to be further analyzed in order to confirm their origin. Overall, these results will significantly enhance the available data for poinsettia in the public databases and will provide useful genetic information that could be exploited for breeding purposes.

### Modulation of bract development

The flowering behavior of plants is regulated by distinct environmental aspects, with light playing a crucial role in several ways. Day-length, or photoperiod, regulates flowering time and allows sexual reproduction to happen at favorable times [73]. Plants are classified according to photoperiodic responses into long-day (LD), in which flowering occurs when the day becomes longer than some crucial length, and short-day (SD), in which flowering occurs when the day becomes shorter [33]. Photoperiod also plays an important role in regulating the biosynthesis of secondary metabolites in plants [34], with longer photoperiods generally promoting anthocyanin biosynthesis [11, 49]. Nonetheless, some plants are able to activate the biosynthesis of anthocyanins in short photoperiod situations. Anthocyanin promotion has been observed in *A. thaliana* due to short photoperiod sensing by phytochrome A [67]. In *Begonia semperflorens*, short-day period, together with low temperatures, is crucial for anthocyanin biosynthesis and it is directly related to increased activities of the enzymes *PAL*, *CHI*, *DFR* and *UFGT* [95].

The flower formation in poinsettia, leading to bract formation and coloration, is induced under short day conditions [41], thus also indicating the role of photoperiodism in anthocyanin induction for the species. The bracts of poinsettia are leaves changing their photosynthetic function into pollinator attraction (i.e. by accumulating anthocyanins) upon flower induction to escort the relatively small and unimpressive reproductive structures [31, 57]. During the bract development process in poinsettia, especially between stages 2 and 3, several photosynthesis related pathways showed a down-regulation in the latest stage, followed by an up-regulation of phenylpropanoid related pathways (Table 4). Increased anthocyanin content levels were detected in the transition from partially to fully pigmented poinsettia bracts, which was accompanied by the reduction of photosynthetic pigments [7, 68]. Moreover, accumulation of chlorophyll was reduced when young poinsettia leaves started to accumulate anthocyanins under short day conditions, which was due to a decrease in the activity of enzymes related to chlorophyll synthesis [36]. In conclusion, the development of poinsettia bracts is marked by a decrease in photosynthesis and chlorophyll biosynthesis genes, followed by increased activity of genes related to flavonoid biosynthesis.

### Regulation of flavonoid pathway between red and white poinsettia varieties during bract development

The anthocyanin biosynthetic pathway is a well-characterized and conserved network in plants, whose regulation is maintained through the expression of structural and regulatory biosynthetic genes [48]. The structural genes can be divided into early biosynthetic genes (EBGs), i.e. *CHS*, *CHI*, *F3H*, *F3’H*, *FLS*, and late biosynthetic genes (LBGs), i.e. *DFR*, *ANS/LDOX*, *UFGT*, *LAR*, *ANR* [22, 59]. EBGs are usually regulated by *R2R3-MYB* regulatory genes, whereas the activation of LBGs is mediated by a regulatory complex, called the MYB-bHLH-WD40 (MBW) complex, consisting of MYB, basic helix-loop-helix (bHLH) and WD40 repeat families [48, 59].

Our gene expression comparison between red and white varieties of poinsettia showed a differential regulation of the flavonoid biosynthesis pathway only at particular stages of bract development. Several structural genes showed a down-regulation on the white variety on the first analyzed stage. Interestingly, two *R2R3-MYB* regulatory genes were also shown to be down-regulated in the white variety: *MYB12* and *MYB113*. *MYB11*, *MYB12,* and *MYB111* from *A. thaliana* share significant structural similarity and are involved in the regulation of the expression of EBGs [59, 70]. In *A. thaliana myb12*-ko mutant seedlings, *CHS* and *FLS* expressions showed a clear reduction, while the expression of *CHI*, *F3H*, *DFR,* and *F3’H* remained unchanged. In contrast, overexpression of *MYB12* in seedlings led to an increased expression of *CHS*, *CHI*, *F3H* and *FLS* [51]. MYB factors have also been demonstrated to positively regulate the expression of EBGs in other species [1, 21, 79, 89].

On the other hand, *R2R3-MYB* factors such as *PAP1*, *PAP2*, *MYB113*, *MYB114* are known to participate in the MBW complex and to regulate the expression of LBGs [10, 28]. In apple, the *MdMYB10* gene, a *MYB113* homologous, showed a positive expression correlation with anthocyanin accumulation, as well as with the expression of LBGs [23]. In *L. formosana*, the *LfMYB113* have been shown to directly activate the expression of two *DFR* homologous, thus promoting the anthocyanin synthesis in leaves [85]. Overexpression of bHLH and MYB-related transcription factor from snapdragon (*Antirrhinum majus*) in tomato fruits resulted in a higher expression of flavonoid-related genes (e.g. *F3’H*, *F3’5’H*, *ANS*, *UFGTs*), thus leading to a higher accumulation of anthocyanins [15].

Our results show an initial impairment of the flavonoid pathway early in the color accumulation process for the white poinsettia variety, but these differences were not observed in the subsequent stages of bract development. In the comparisons between red and white varieties for stages 2 and 3, most of the previously down-regulated genes related to flavonoid biosynthesis did not show any differential expression. In fact, some of them showed an up-regulation in the white variety for those stages; however, a few of these genes contain multiple annotated transcripts (e.g. *CHS* and *FLS*) with different expression patterns. In fact, *CHS* has been shown to play a major role in anthocyanin biosynthesis in different species, in which the appearance of white flowers or flower segments is driven by a lack of its expression [26, 53, 58, 71]. CHS, a well-characterized enzyme with a key role in the early steps of flavonoid biosynthesis, is known to be encoded by a multigene family in many plant species [81, 88]. In turnip, six *CHS* genes were identified, but only three of them were shown to be functional and to promote anthocyanin biosynthesis [100]. Three *CHS* genes have been characterized in *Citrus* and they have been shown to contribute differently and complementarily to the production of flavonoids [82]. Two out of four *CHS* identified in our bract transcriptome showed a differential expression between red and white varieties. However, this does not seem to affect the overall functionality of the flavonoid pathway in the poinsettia varieties analyzed in our study, since the pigmentation of bracts is due to the accumulation of flavonoid compounds [69]. Taking all together, the initial impairment observed for the flavonoid pathway does not seem to be responsible for the lack of anthocyanin accumulation in white poinsettia bracts. This is confirmed by the constitutive expression of EBGs and LBGs in stages 2 and 3 of bract development.

The last step of the anthocyanin biosynthesis is characterized by the transfer of the glucosyl moiety from UDP-glucose to the 3-hydroxyl group of anthocyanidins by *UDP glucose: flavonoid 3-*O*-glucosyltransferase* (*UFGT*), which results in the formation of stable colored pigments of anthocyanins 3-*O*-glucosides, as well as providing stability and water solubility in the plant [92, 99]. *UFGT* expression has been positively linked with anthocyanin accumulation in grapes and apples [39, 52]. In *A. thaliana*, *UGT78D2* (At5g17050) and *UGT75C1* (At4g14090) are the main genes suggested to be involved in the modification of the sugar moieties of anthocyanins, but with *UGT79B1* (At5g54060) having similar functions [42, 75, 91]. In our dataset, we identified a *UGT79B10* gene being up-regulated in the red variety at stage 3, which is highly similar to the *UGT79B1* gene and, therefore, might be also involved in the anthocyanin formation in poinsettia.

After biosynthesis, most conjugated flavonoids are transported and deposited primarily to the vacuole [45, 86], where vacuolar pH and the presence of co-pigments determine anthocyanin-mediated coloration [98]. Three distinct mechanisms for flavonoid transport in plant cells have been proposed: vesicle trafficking, membrane-mediated transport, and Glutathione S-transferase (GST) mediated transport [98]. GST genes play an important role in anthocyanin transportation, since GST mutants show phenotypes with a visible lack of pigmentation, such as *bz2* (*Bronze-2*) from maize, *an9* (*Anthocyanin 9*) from petunia, *tt19* (*Transparent Testa 19*) from Arabidopsis and *fl3* (*Flavonoid3*) from carnation [4, 38, 44, 50]. Moreover, there is a high conservation of GSTs involved in flavonoid accumulation [97] and, therefore, they are able to complement each other’s expression.

In our differential expression analysis, a *GSTF11* Arabidopsis homolog gene showed a higher expression in the red variety for the last stage of bract development for both RNA-Seq and RT-qPCR analyses (Fig. 4). Although *GSTF12* is shown to be involved in anthocyanin transport [38], they share a high similarity. In fact, the poinsettia putative *GST* gene shares 58 and 55% amino acid identity with Arabidopsis *GSTF11* and *GSTF12*, respectively, which is higher than between Arabidopsis *TT19* and petunia *AN9* (50% amino acid identity) [38]. Due to its homology to known anthocyanin-related GSTs, the putative poinsettia *GST* is a promising candidate for white coloration in poinsettia.

## Conclusions

In this study, we provide a comprehensive hybrid transcriptome from poinsettia bracts. In addition, we provide for the first time a profiling of gene expression during the process of bract development of red and white poinsettia varieties. Our differential expression analysis revealed that the majority of the anthocyanin-related genes are equally expressed in red and white varieties. Nonetheless, *UGT79B10* and *GSTF11* showed a lower expression in the last stage of bract development for the white variety, which are involved in glucosylation and transport of anthocyanins. The role of the putative *UGT79B10* and *GST* in the differences in anthocyanin accumulation in red and white poinsettias is still unknown. Functional studies are needed in order to clarify their possible role in the transition from red to white bracts. Nonetheless, these genes*,* and genes regulating their expression, are potential candidates for further studies.

Our transcriptome analysis provides a valuable foundation for further studies on the species, such as plant breeding and genetics, and highlights crucial information on the molecular mechanism of color formation in poinsettia. It should promote further investigations into the detailed regulatory pathways regulating flavonoid biosynthesis and contribute to a better understanding of the *white paradox* in the species.

## Methods

### Plant material and growth conditions

**Fig. 5:**
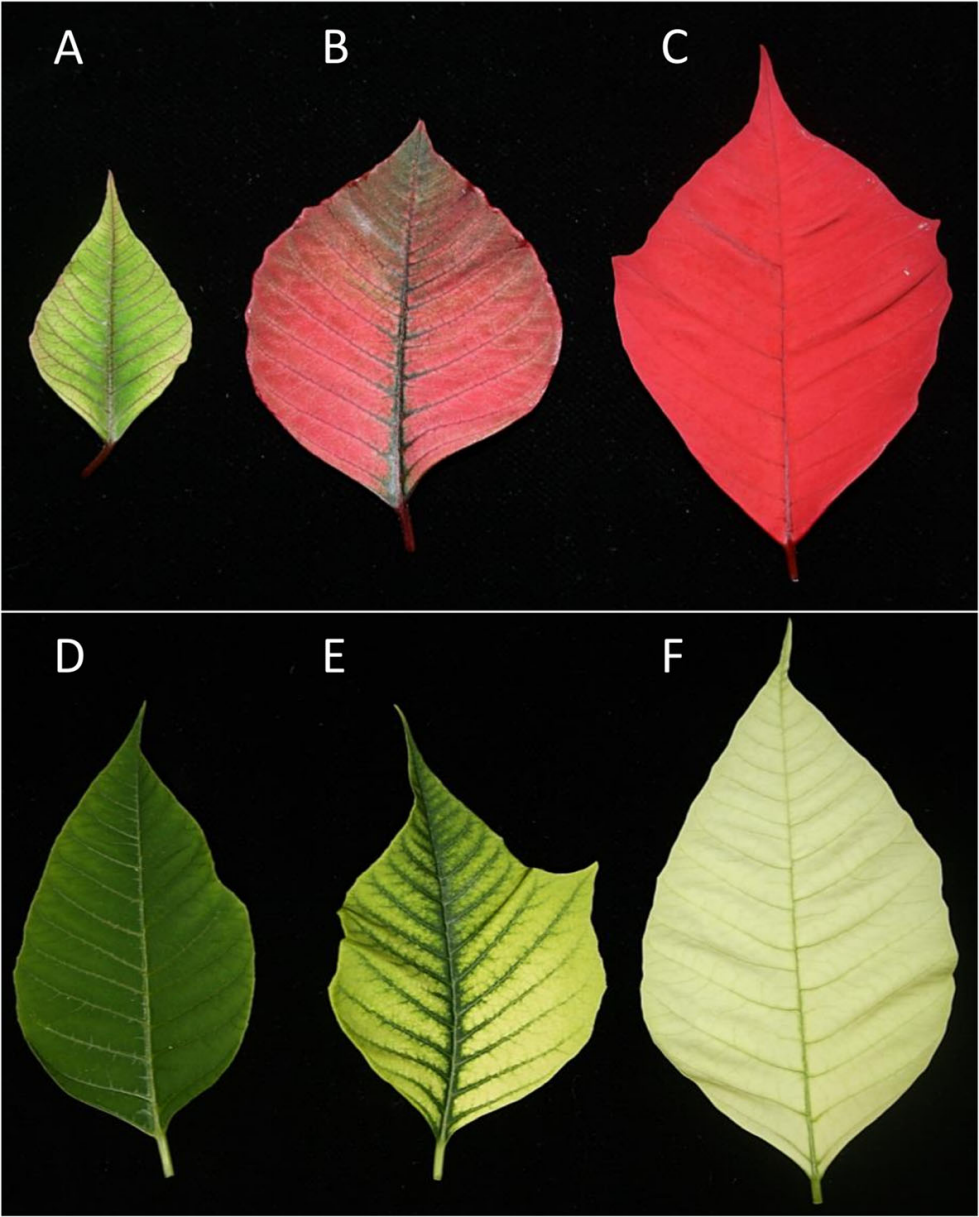
Bracts of red and white poinsettia varieties for three color developmental stages. **a-c** Bracts from the Christmas Feelings variety for stages 1, 2 and 3, respectively; **d-f** Bracts from the Christmas Feelings Pearl variety for stages 1, 2 and 3, respectively

The red bracted poinsettia varieties Vintage and Christmas Feelings, as well as the white bracted variety Christmas Feelings Pearl were used in the present study. The white variety was obtained through radiation mutagenesis of the red variety, followed by shoot development and trait selection at the company Selecta One (Stuttgart, Germany). Therefore, red- and white-bracted poinsettias from the same variety are referred to as ‘pairs’, due to their highly similar genetic background. The varieties’ names, bract color, number of biological replicates and other aspects are shown in Table 5. Bract samples were harvested in three color developmental stages: i) Stage 1 (S1) – defined as the transition of green colored leaves to red/white colored bracts, with the majority of the bracts still bearing a greenish coloration; ii) Stage 2 (S2) – defined as the presence of both green and red/white colors in the bracts, with a major part of the bracts bearing red/white coloration; and iii) Stage 3 (S3) – defined as a fully developed red/white coloration, with no major green coloration visible on the bracts. For a visual representation of the stages, please refer to Fig. 5.

**Table 5:** Pairs of red and white poinsettia varieties used in the present study

Type of analysis	Variety name	Bract coloration	Color stage	# of biological replicates
Illumina RNA-Seq *single-end*	Christmas Feelings	Red	S1	3
	Christmas Feelings Pearl	White	S1	3
	Christmas Feelings	Red	S2	3
	Christmas Feelings Pearl	White	S2	3
	Christmas Feelings	Red	S3	3
	Christmas Feelings Pearl	White	S3	3
Illumina RNA-Seq *paired-end*	Christmas Feelings	Red	S3	1
	Christmas Feelings Pearl	White	S3	1
PacBio RNA-Seq	Vintage	Red	S3	1
RT-qPCR	Christmas Feelings	Red	S1	5
	Christmas Feelings Pearl	White	S1	5
	Christmas Feelings	Red	S2	5
	Christmas Feelings Pearl	White	S2	5
	Christmas Feelings	Red	S3	5
	Christmas Feelings Pearl	White	S3	5

Rooted cuttings from all varieties were obtained from Selecta One company (https://www.selecta-one.com/) and grown in a greenhouse, at the Institute for Plant Genetics from the Leibniz Universität Hannover (Hannover, Germany), under short-day conditions to induce flower formation and to stimulate the development of colored bracts. The plants were grown in 5 L pots containing Einheitserde P substrate (Hermann Meyer KG, Germany), with an average temperature of 22 °C and 9 h of daylight (15 h of darkness). Bract samples were harvested, immediately frozen in liquid nitrogen and stored at − 80 °C for subsequent analysis.

### Tissue sampling, RNA isolation, and quantification

Bract samples from all varieties used for RNA-Seq were sent on dry ice to vertis Biotechnologie AG (Freising, Germany) for processing. Total RNA was isolated from approximately 100 mg of bract tissue using the mirPremier™ miRNA isolation kit (Sigma-Aldrich, St. Louis, USA) according to the manufacturer’s instructions. Total RNA samples were analyzed for integrity by capillary electrophoresis using Shimadzu MultiNA microchip electrophoresis MCE-202 MultiNA Microchip Electrophoresis System (Shimadzu Corp., Kyoto, Japan).

For RT-qPCR analysis, total RNA was isolated from approximately 100 mg of bract tissue using the mirPremier™ miRNA isolation kit (Sigma-Aldrich) at the Institute for Plant Genetics from the Leibniz Universität Hannover. The total RNA concentration was analyzed using NanoDrop™ 2000 (Thermo Fisher Scientific, Wilmington, USA) and agarose gel electrophoresis.

### PacBio sequencing and data processing

A full-length cDNA library from the Vintage variety was prepared at vertis Biotechnologie AG. Briefly, Poly(A) + RNA was isolated from the total RNA sample and the 5’CAP structure was removed using CAP-Clip™ Acid Pyrophosphatase (Cellscript, Wisconsin, USA). Afterward, an RNA adapter was ligated to the 5′-monophosphate of the RNA. First strand cDNA was synthesized using an oligo (dT)-linker primer and M-MLV [H–] Reverse Transcriptase (Promega, Wisconsin, USA). The library sequencing was performed at the Vienna BioCenter Core Facilities GmbH (Vienna, Austria) using the PacBio Sequel System based on the Single Molecule, Real-Time (SMRT) Sequencing technology.

The Isoform Sequencing (Iso-Seq) Analysis v3.0 pipeline (https://github.com/ben-lerch/IsoSeq-3.0) was used to analyze the PacBio dataset. The pipeline was performed in three stages: i) CCS, where circular consensus sequences (CCS) were built from subreads; ii) Classify, where CCSs were classified as full-length non-chimeric (FLNC) reads and non-full length (NFL) reads; and iii) Cluster, where the sequences were clustered in high-quality consensus sequences (contigs).

### Illumina sequencing and data processing

Two different sequencing strategies were used for the Illumina sequencing. In the first one, 1x75bp *single-end* 3′ cDNA libraries were constructed for the varieties Christmas Feelings and Christmas Feelings Pearl for the different bract developmental stages. Poly(A) + RNA was isolated from the total RNA samples and the first-strand cDNA was synthesized using an oligo (dT)-adapter primer and M-MLV reverse transcriptase. After fragmentation, the first-strand cDNA was purified, the 5′ Illumina TruSeq sequencing adapter was ligated to the 3′ end of the antisense cDNA and, finally, amplified by PCR.

For the second strategy, 2x150bp *paired-end* cDNA libraries were constructed for the varieties Christmas Feelings and Christmas Feelings Pearl for the third stage of bract development (S3). Ribosomal RNA molecules were depleted using the Ribo-Zero rRNA Removal Kit for plants (Illumina, San Diego, USA). Second, the first-strand cDNA was synthesized using an N6 randomized primer. After fragmentation, the Illumina TruSeq sequencing adapters were ligated in a strand-specific manner to the 5′ and 3′ ends of the cDNA fragments and the cDNA was finally amplified by PCR. Both *paired-end* and *single-end* libraries were sequenced at vertis Biotechnologie AG using an Illumina NextSeq500 system.

Reads representing ribosomal RNA gene fragments (rRNAs) were removed from the datasets using the sortmerna tool v2.1 [40] with all included databases: SILVA and Rfam [35, 63]. Reads were trimmed and filtered using Trimmomatic v0.36 [14] with the parameters adapted to both sequencing strategies: 2x150bp *paired-end*: TRAILING:20 AVGQUAL:20 SLIDINGWINDOW:5:20 MINLEN:75; 1x75bp *single-end*: TRAILING:20 AVGQUAL:20 SLIDINGWINDOW:5:20 MINLEN:50.

### Transcriptome assembly, annotation, and completeness of the transcriptome

The poinsettia bract transcriptome was assembled using the high-quality PacBio consensus sequences and the 150 bp *paired-end* processed Illumina reads from Christmas Feelings and Christmas Feelings Pearl varieties. The assembly was performed with Trinity v2.7.0 [29] using the long-reads assembly option. The transcriptome was annotated by sequence similarity against the *Arabidopsis thaliana* genome (TAIR10 protein representative gene model) [12] and the SwissProt databases [77] using BLASTX v2.8.0 (*E-value* < 1E-20) [16]. GO terms were retrieved, for the final poinsettia bract transcriptome, from the best hits obtained from BLASTX against the TAIR10 database using the online tool agriGO v2.0 [74]. Additionally, the BUSCO pipeline v1.2 [83] with its plant set (embryophyta_odb9) was used to assess the completeness of the poinsettia bract transcriptome.

For understanding the sequence and quantitative differences between tissue-specific transcripts in *Euphorbia* species, short *paired-end* Illumina RNA sequences from *Euphorbia pekinensis* root, stem, and leaf tissues were retrieved from the NCBI Sequence Read Archive (SRA) Sequence Database (accession number SRP097008) [18]. Ribosomal RNAs were removed from the datasets using the sortmerna tool v2.1 [40], followed by low-quality reads (average quality score below 20) trimming using Trimmomatic v0.36 [14] with the parameters TRAILING:20 AVGQUAL:20 SLIDINGWINDOW:5:20 MINLEN:75. De novo transcriptomes were assembled for each of the tissues using Trinity v2.7.0 [29]. Annotation and retrieval of GO terms for each of the tissues’ transcriptomes were done in a similar way as for the poinsettia transcriptome.

### Differential gene expression and pathway enrichment analysis

Illumina processed reads from the different red and white poinsettia samples were used for the differential gene expression (DGE) analysis. Transcript abundance quantification was performed with the RSEM tool [46] and bowtie2 was selected as the alignment method. Low expressed transcripts (Counts Per Million (CPM) ≤ 0.5 in at least 2 biological replicates) were removed from the dataset. Normalizations and pair-wise comparisons were performed with edgeR [65]. The thresholds for a differentially expressed gene (DEG) were set as: i) False Discovery Rate (FDR) ≤ 0.05; ii) log2FC ≥ 1 or ≤ − 1; and iii) Fragments Per Kilobase of transcript per Million mapped reads (FPKM) ≥ 1.0 for three biological replicates in at least one of the compared stages.

The differentially expressed genes for each of the comparisons were subjected to Single Enrichment Analysis (SEA) using the online tool agriGO v2.0, with the following parameters: 1) Selected species: *Arabidopsis thaliana*; 2) Reference: TAIR genome locus (TAIR10_2017); 3) Statistical test method: Hypergeometric; 4) Multi-test adjustment method: Hochberg (FDR); 5) Significance level of 0.05; 6) Minimum number of 5 mapping entries; and 7) Gene ontology type: Complete GO.

### Quantitative PCR

cDNA synthesis was performed using the FastGene Scriptase Basic cDNA Kit (Nippon Genetics Europe GmbH, Düren, Germany) according to the manufacturer’s recommendations. A total of five independent biological replicates were used for each of the varieties and stages analyzed (Table 5). The RT-qPCRs were performed using the qPCRBIO SyGreen Mix Lo-ROX kit (Nippon Genetics Europe GmbH) according to the manufacturer’s recommendations. Briefly, reactions were carried out in technical triplicates in a volume of 10 μL containing 5 μL of qPCRBIO SyGreen Mix Lo-ROX, 10 μmol of gene-specific forward and reverse primers, and 4 μL of 1:50 cDNA dilution. RT-qPCRs were performed using a StepOne™ Real-Time PCR System (Applied Biosystems, Singapore, Singapore). The normalized relative quantity (NRQ) was calculated according to the Pfaffl equations [60]. Two reference genes (Translation elongation factor 1 beta – *EF1B*; and Translation elongation factor 1 alpha – *EF1A* [94]) were used to normalize the expression data. The list of genes and primer sequence-design for the RT-qPCR reactions are available in Additional file 12. Statistical analysis was performed using the Relative Expression Software Tool (REST) v2.0.13 [60].

## Supplementary information

**Additional file 1. **Histogram of average coverage of *paired-end* Illumina reads from Christmas Feelings (A) and Christmas Feelings Pearl (B) varieties mapped to the 30,768 PacBio contigs.

**Additional file 2.** Annotation results of the poinsettia bract transcriptome against the TAIR10 database.

**Additional file 3.** Distribution of percent length coverage for the top matching database entries from the poinsettia bract transcripts.

**Additional file 4. **BUSCO results for the *E. pulcherrima* and *E. pekinensis* transcriptomes.

**Additional file 5.** Summary of the processed libraries for the Christmas Feelings and Christmas Feelings Pearl poinsettia varieties in three bract developmental stages.

**Additional file 6.** Pearson correlation for the biological replicates of Christmas Feelings and Christmas Feelings Pearl paired-end Illumina datasets for the three stages of bract development in poinsettia.

**Additional file 7.** Differentially expressed transcripts for the comparisons between bract developmental stages in poinsettia.

**Additional file 8.** List of enriched GO terms for the comparisons between bract developmental stages in poinsettia.

**Additional file 9.** List of transcripts present in the differentially enriched pathways related to photosynthesis and phenylpropanoid metabolic processes.

**Additional file 10.** Differentially expressed transcripts for the comparisons between Christmas Feelings (red) and Christmas Feelings Pearl (white) poinsettia varieties for the different bract developmental stages.

**Additional file 11.** List of enriched GO terms for the comparisons between Christmas Feelings (red) and Christmas Feelings Pearl (white) poinsettia varieties for the different bract developmental stages.

**Additional file 12.** Primer sequences for each of the genes analyzed by RT-qPCR in poinsettia bracts.

## Data Availability

The full sequencing dataset (Illumina and PacBio) is available through the Sequence Read Archive (SRA) at NCBI under BioProject number PRJNA532349.

## Abbreviations

CCS: Circular consensus sequences
cDNA: Complementary DNA
CPM: Counts Per Million
DEG: Differentially expressed gene
DGE: Differential gene expression
DNA: Deoxyribonucleic acid
EBG: Early biosynthetic gene
FDR: False discovery rate
FLNC: Full-length non-chimeric reads
FPKM: Fragments Per Kilobase of transcript per Million mapped reads
GC content: Guanine-cytosine content
GO: Gene ontology
GST: Glutathione S-transferase
LBG: Late biosynthetic gene
LD: Long-day
MBW complex: MYB-bHLH-WD40 complex
NCBI: National Center for Biotechnology Information
NFL: Non-full length reads
NRQ: Normalized relative quantity
PCR: Polymerase chain reaction
PPR: Pentatricopeptide repeat
RNA: Ribonucleic acid
RNA-Seq: RNA sequencing
ROS: Reactive oxygen species
rRNA: Ribosomal RNA
RT-qPCR: Quantitative reverse transcription PCR
SD: Short-day
SMRT: Single Molecule, Real-Time Sequencing technology
SRA: Sequence Read Archive
