# Supplementary material for: Hybrid de novo transcriptome assembly of poinsettia (Euphorbia pulcherrima Willd. Ex Klotsch) bracts
Source: BMC Genomics. 2019 Nov 27;20:900. doi: 10.1186/s12864-019-6247-3 (PMC6882326; doi:10.1186/s12864-019-6247-3)
Supplement: Supplementary file 1 — Additional file 1. Histogram of average coverage of paired-end Illumina reads from Christmas Feelings (A) and Christmas Feelings Pearl (B) varieties mapped to the 30,768 PacBio contigs. [file 12864_2019_6247_MOESM1_ESM.docx]

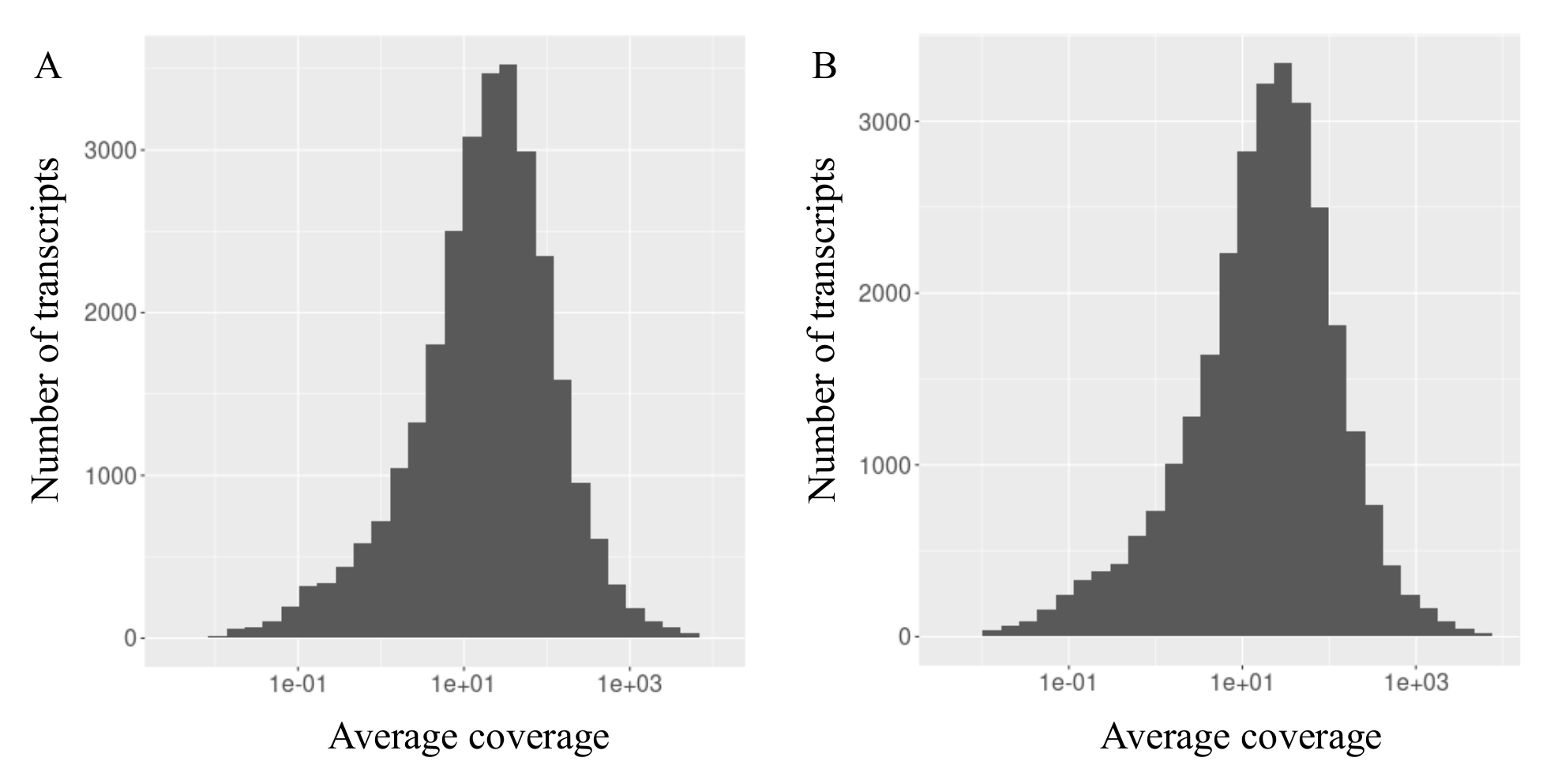


**Additional File 1.** Histogram of average coverage of *paired-end* Illumina reads from Christmas Feelings (A) and Christmas Feelings Pearl (B) varieties mapped to the 30,768 PacBio contigs.
