# Supplementary material for: Hybrid de novo transcriptome assembly of poinsettia (Euphorbia pulcherrima Willd. Ex Klotsch) bracts
Source: BMC Genomics. 2019 Nov 27;20:900. doi: 10.1186/s12864-019-6247-3 (PMC6882326; doi:10.1186/s12864-019-6247-3)
Supplement: Supplementary file 6 — Additional file 6. Pearson correlation for the biological replicates of Christmas Feelings and Christmas Feelings Pearl paired-end Illumina datasets for the three stages of bract development in poinsettia. [file 12864_2019_6247_MOESM6_ESM.docx]

**Additional File 6.** Pearson correlation for the biological replicates of Christmas Feelings and Christmas Feelings Pearl *paired-end* Illumina datasets for the three stages of bract development in poinsettia.


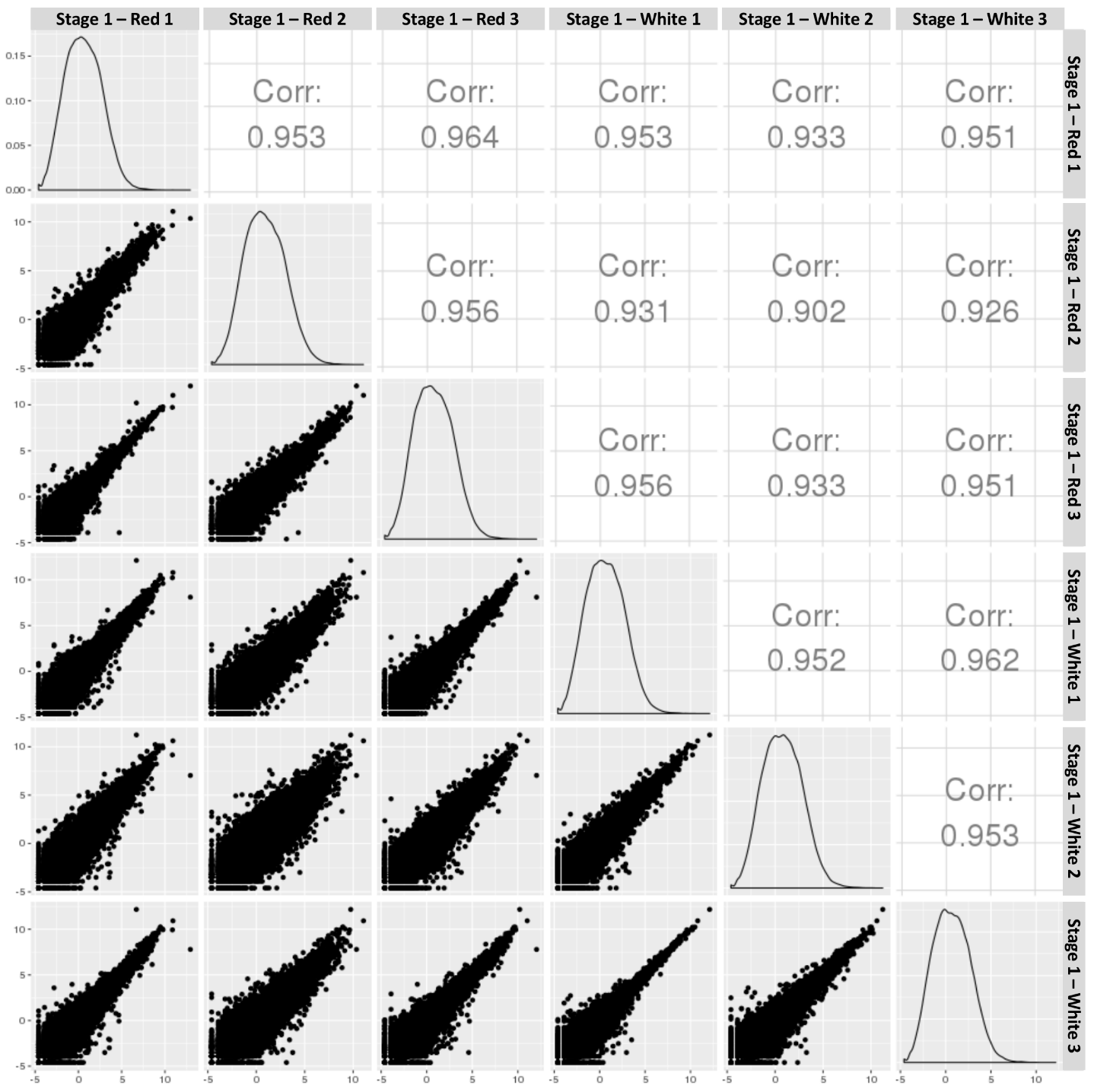


**Figure 1.** **Pearson correlation for the biological replicates of Christmas Feelings and Christmas Feelings Pearl for the first stage of bract development.** Red 1, 2 and 3 = Biological replicates 1, 2 and 3 from the variety Christmas Feelings. White 1, 2 and 3 = Biological replicates 1, 2 and 3 from the variety Christmas Feelings Pearl.


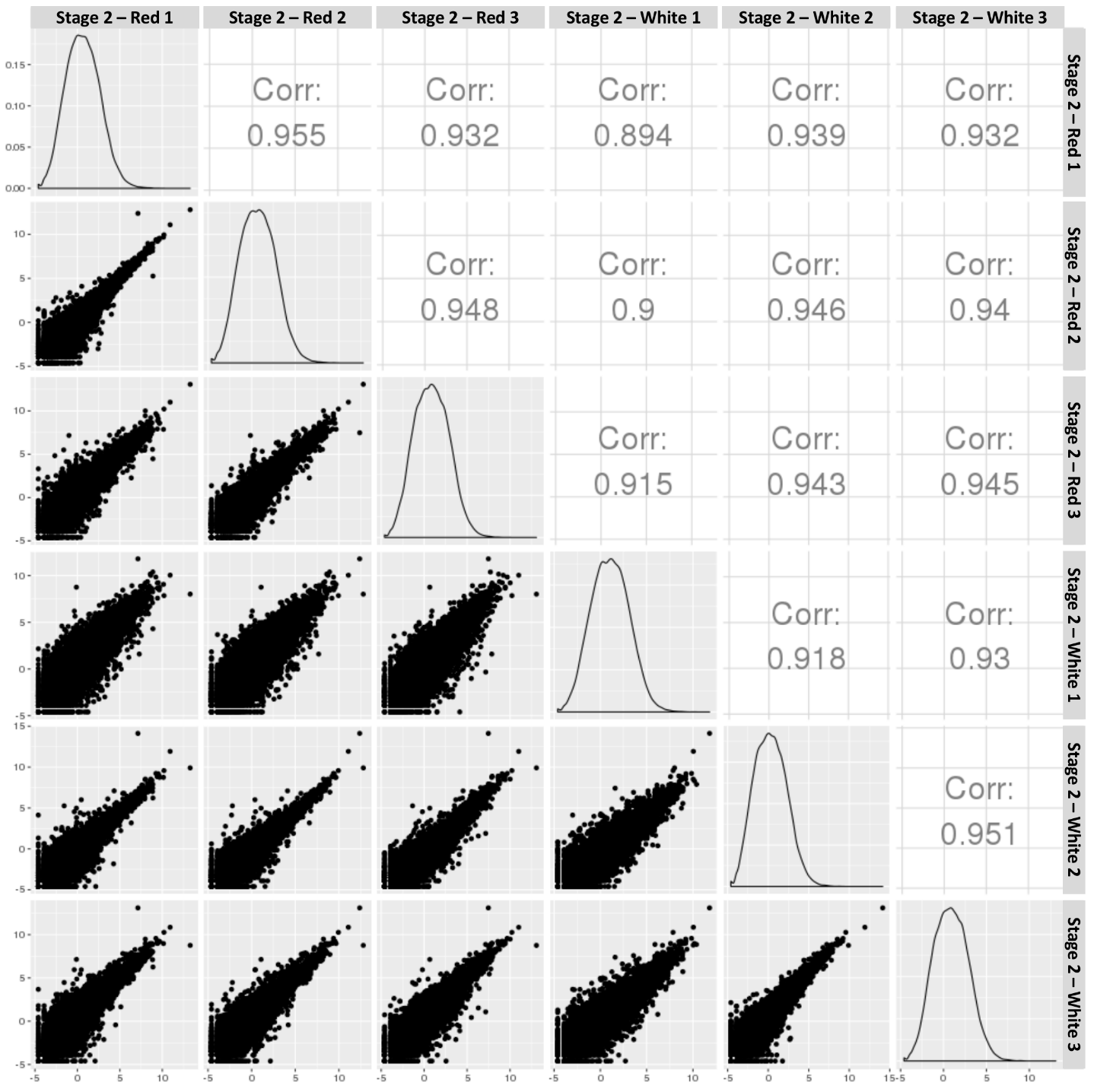


**Figure 2.** **Pearson correlation for the biological replicates of Christmas Feelings and Christmas Feelings Pearl for the second stage of bract development.** Red 1, 2 and 3 = Biological replicates 1, 2 and 3 from the variety Christmas Feelings. White 1, 2 and 3 = Biological replicates 1, 2 and 3 from the variety Christmas Feelings Pearl.


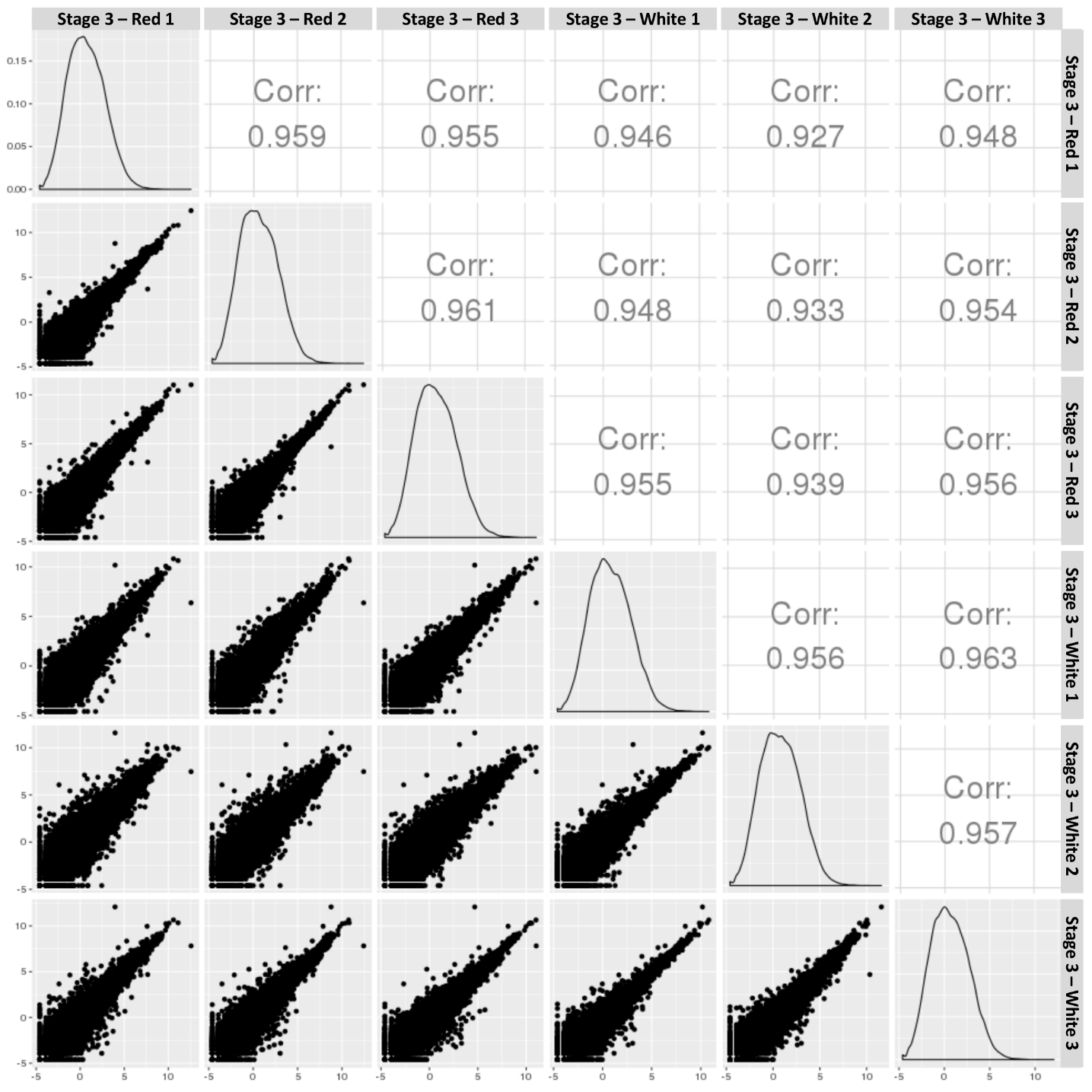


**Figure 3.** **Pearson correlation for the biological replicates of Christmas Feelings and Christmas Feelings Pearl for the third stage of bract development.** Red 1, 2 and 3 = Biological replicates 1, 2 and 3 from the variety Christmas Feelings. White 1, 2 and 3 = Biological replicates 1, 2 and 3 from the variety Christmas Feelings Pearl.
